# Validity and reliability of the European Organization for Research and Treatment in Cancer Quality of Life Questionnaire (EORTC QLQ): experience from Kuwait using a sample of women with breast cancer

**DOI:** 10.4103/0256-4947.67083

**Published:** 2010

**Authors:** Shafika A. Alawadhi, Jude U. Ohaeri

**Affiliations:** a Faculty of Medicine, Kuwait University, Safat, Kuwait; b Department of Psychiatry, Psychological Medicine Hospital, Safat, Kuwait

## Abstract

**BACKGROUND AND OBJECTIVES:**

Although the EORTC QLQ-C30 and its breast-specific module (BR-23) are widely used instruments, the few reports on their psychometric characteristics from Arab and neighboring countries involved limited analyses. Our objective was to assess the psychometric characteristics of both questionnaires using the responses of a larger sample of Arab women.

**METHODS:**

Participants were consecutive clinic attendees at the Kuwait Cancer Control Center. The indices assessed were alpha coefficients, item-internal consistency (IIC), item-discriminant validity (IDV), and known-groups validity.

**RESULTS:**

The 348 women were aged 48.3 (10.3) years. The intra-class correlation for the test-retest statistic and the internal consistency values for the multi-item scales were >0.7 alpha. With the exception of the pain subscale, all items met the IIC criterion of >0.4 correlation with the corresponding scale. For IDV, the BR-23 performed better than the QLQ-C30. The scale scores discriminated between patients at different disease stages, and between sick and well populations.

**CONCLUSION:**

With the exception of the pain subscale, the Arabic version of the questionnaires is psychometrically sound.

The effectiveness of modern methods of treatment and detection in oncology has contributed to the popular interest in quality of life (QOL) issues among survivors.^1^ Although there has been considerable research on the QOL of women with breast cancer,^2^ there is a paucity of such reports from the Arab and neighboring countries.^3^–^6^ Research interest in this field in the Arab world will be facilitated by the availability of psychometrically sound disease-specific instruments that have cross-cultural applicability. Towards this end, the European Organization for Research and Treatment in Cancer Quality of Life Questionnaire (EORTC QLQ-C30)^7^ and its breast-specific module (BR-23)^8^ are useful because they have been validated in diverse cultures,^2^ and an official Arabic translation exists.

Previous reports on the validity and reliability of the EORTC QLQ-C30 and BR-23 from the Arab and neighboring countries^3^,^5^,^6^,^9^ have been based on relatively small sample sizes and the analyses were limited to a few issues of psychometrics. For instance, multi-trait scaling and test-retest reliability analyses were limited, and known-groups validity analysis did not involve comparison with a general population group.^10^

Our specific objectives were to assess (i) the test-retest reliability of the EORTC QLQ-C30 and BR-23; (ii) the floor/ceiling effect and acceptability of the items, as well as the internal consistency of the full questionnaires and their constituent multi-item scales; (iii) The item-internal consistency (IIC) and item-discriminant validity (IDV);^11^ and (iv) the known-groups validity.^10^

## METHODS

The subjects were consecutive attendees at the outpatient clinic of the medical oncology department of the Kuwait Cancer Control Center (KCCC) who fulfilled the study’s inclusion criteria. They were attending follow-up clinic appointment for chemotherapy. Participants were in stable clinical condition and could independently give consent to participate in the study.^7^ In this culture, female patients are, as a rule, accompanied to hospital by family members who live with them.^12^ Consent was also obtained from the family members, but the patients were interviewed privately in one of the clinic rooms. The KCCC is the national center for treatment of cancer. It has adequate facilities for diagnosis and treatment of cancer.

The EORTC QLQ-C30 is a 30-item generic health-related QOL instrument designed to assess cancer patients’ physical, psychological and social functioning.^7^,^13^ It is composed of nine multi-item scales (5 functional scales, a global QOL scale [GQOL], and three symptom scales), five single-item symptom scales, and an item on the perceived financial impact of the disease (Table 1). In the version used for this study (version 3), the first 28 items are rated on a response scale of “not at all” (1), to “very much” (4).

**Table 1:** Reliability analyses for QLQ C-30 and BR-23.

Variables/subscales	No. of subjects	No. of items	Cronbach’s aplha	Floor effect (%) (range)	Ceiling effect (%) (range)
QLQC 30 for first 28 items	253	28	0.94^a^		
Physical functioning	330	5	0.79	3.3‐14.4	7.5‐20.9
Emotional	323	4	0.83	4.7‐6.0	16.4‐26.4
Fatigue	331	3	0.76	2.9‐6.5	14.4‐24.9
Body image	299	4	0.85	3.9‐7.7	21.1‐25.4
Systemic side effects	300	7	0.79	3.0‐14.2	15.6‐28.1
BR-23	171	23	0.92^b^		
Breast symptoms	293	4	0.88	4.8‐6.0	23.2‐35.0
Arms symptoms	318	3	0.76	6.2‐7.3	21.8‐28.7
Role functioning	342, 344	2		5.2‐5.3	10.9‐12.9
Cognitive	343, 345	2		5.0‐5.5	18.4‐24.3
Social functioning	341	2		5.0‐5.3	16.7‐22.9
Nausea and vomiting	340	2		2.4‐3.2	33.5‐40.3
Pain	338, 343	2		5.2‐5.7	13.6‐15.2
Dyspnoea	335			6.0	18.8
Insomnia	338			7.1	18.3
Appetite loss	339			4.7	25.1
Constipation	345			3.5	42.0
Diarrhea	343			1.7	54.8
Financial difficulties	344			2.9	29.1
Global QOL	340			1.2, 1.5	1.8
Sexual function	308, 326			0.6, 1.5	31.3, 37.3
Sexual enjoyment	221			2.3	16.7
Future perspective	334			11.7	25.4
Upset by hair loss	331			10.9	17.2
Test-retestdata^c^					
1st test:QLQ C-30 for first 28 items	77	28	0.93		
Test-retest for first 28 items	67	56	ICC:0.97 (0.95‐0.98)		
2nd test: first 28 items of C-30	79	28	0.94		
Test-retest for general health and global QOL	92	4	ICC:0.93 (0.89‐0.95)		
1st test BR-23	37	23	0.91		
Test-retest for BR-23	27	46	ICC: 0.94 (0.90‐0.97)		

a Split-half reliability=0.84;

b Split-half reliability=0.81.

c Test-retest data: Mean age=31(7.6), range 23‐65 years, median=28, mode=24, N=95.

The 23-item breast cancer-specific module, the QLQ-BR-23,^8^ consists of two multi-item functional scales, three multi-item symptom scales, and three single-item scales (Table 1). The response options are similar. The scoring algorithm recommended by the EORTC^14^ was used to transform the responses to values on a scale of 0% to 100%. For the functional scales and GQOL, a higher score corresponds to better functioning and QOL. For symptom scales, a higher score corresponds to more frequent and/or more intense symptoms.

The EORTC Quality of Life Unit in Belgium kindly sent us the questionnaires (English and Arabic translations). Ethical approval for the work was obtained from the institutional review panel of the KCCC. In addition, patients and their family caregivers gave verbal informed consent to participate in the study. They were duly informed that there would be no negative consequences for declining to participate. All families approached freely consented to participate in the study.

All assessments were based on private interviews by a trained female Arab research assistant. The criteria for staging disease by the doctors were those of the American Joint Committee on Cancer.^15^ Test-retest reliability was done by giving the questionnaires twice in a one-week period to 95 randomly selected literate, healthy, Kuwaiti women (aged >20 years, and married, to match the patients’ socio-demographic characteristics).

Data were analyzed by SPSS, version 15 (SPSS Inc., Chicago, Illinois). The scale scores of the QLQ-C30 and BR-23 were computed as recommended.^14^ Data for test-retest reliability were analyzed by intra-class correlation coefficient (ICC), Kedall’s tau correlation and kappa statistic for item agreements.^16^ The internal consistency was assessed by Cronbach’s alpha. Acceptability of the questionnaires was assessed by the proportion of respondents who failed to complete each item. A cut-off value of <2.5% is recommended.^11^ The proportion of respondents scoring at the lowest level (i.e., floor effect) and the highest level (ceiling effect) for each item was assessed. This is a measure of how far the item can be assumed to be capturing the full range of potential responses in the population.^11^ Item internal consistency (IIC) and item discriminant validity (IDV), measured by Pearson’s correlation, were assessed after adjusting for item overlap in the corresponding scale. The IIC and IDV concern the relationship of each item to its hypothesized scale or domain. The IIC rule requires that the item should correlate r ≥0.4 with its adjusted scale score. For IDV, the item should have the highest correlation with its scale, in comparison with other scales in the questionnaire.^11^

Known-groups validity was assessed, first by testing the significant differences in scale scores between subjects at different stages of the disease. Second, we compared scale score differences between the patients and the general population group, by effect size calculations and by adopting the operational definition of a clinically meaningful (significant) difference of 10% between groups.^17^ For effect size calculations, we defined a clinically significant difference as ≥ 0.5.^18^ Missing data were automatically handled by the SPSS program by excluding cases analysis-by-analysis. The level of statistical significance was set at 5%.

## RESULTS

In 2007 and 2008, 348 women fulfilled the inclusion criteria and agreed to participate. They were aged 20 to 81 years, with a mean and standard deviation of 48.3 (10.3) years. Six subjects (2.1% of 345) were aged <30 years, while 82 (23.8%) were aged >55 years. The majority (58.7%) were being treated for advanced disease (i.e., stages III and IV). The general population sample consisted of 95 women, aged 31(7.6) years (range: 23 to 55 years).

### Reliability

The internal consistency values for the full questionnaires and their multi-item scales (i.e., ≥ 3 items) met the 0.7 Cronbach’s alpha value requirement for the responses of the patients (Table 1). With regard to the floor and ceiling effects for the items of the QLQ-C30, the frequency of lowest scores was 0.6% to 14.4%, while the frequency of highest scores was 1.8% to 54.8%. For the QLQ-C30, 23 (76.7%) items had <2.5% missing values. For the BR-23, items from the following subscales had missing values: sexual (>6.3%), breast (>4.6%), body image (>3.2%), and upset by hair loss (9.2%).

The mean scores for the first 28 items of the QLQ-C30 (possible score: 1-4) ranged from 2.0 (0.75) to 2.73 (0.79) for 23 items, and for the two global QOL items (possible range: 1-7), it was 3.68-3.76. For the BR-23, the mean scores for 21 items (possible range: 1-4) was 2.06 (0.61) to 2.46 (0.95). The ICC for the test-retest statistic (general population data) was highly significant for both the QLQ-C30 (0.97) and the BR-23 (0.94) (Table 1). Accordingly, the Kendall tau correlation coefficients for items in the test-retest data for the QLQ-C30 were high (all were >0.60, except five items with 0.5-0.59). In addition, the Kappa values indicated that all agreements were at least moderate (i.e., k>0.41), with 10 being substantial (i.e., k>0.61, *P*<.0001). A similar result was noted for the BR-23.

### Item internal consistency and item discriminant validity

All the 15 items of the functional scales of the QLQ-C30 met the IIC requirement of correlation ≥0.4 (Table 2). With regard to IDV, there were definite scaling errors (see Table 2 for definition) for the following: (i) the two items of role functioning; and (ii) the two items of cognitive functioning. However, these scaling errors were conceptually logical because the items correlated with scales of similar construct (e.g., limitations in role functioning correlated with physical health). Similarly, in those items with probable scaling error (see Table 2 for definition), the correlations were conceptually logical.

**Table 2:** Item-internal consistency and item-discriminant validity for QOLQ- C30.

Scale items	Physical (1)	Role (2)	Emotional (3)	Cognitive (4)	Social (5)
(1) Difficult effort	0.55	0.48	0.35	0.43	0.47
Problem walking long	0.61	0.49	0.29	0.35	0.39
Walking short	0.61	0.49	0.34	0.45	0.29
Need to stay in bed^a^	0.52	0.55	0.27	0.29	0.23
Need help eating	0.54	0.61	0.39	0.43	0.26
(2) Restrcited doing work^b^	0.59	0.43	0.41	0.43	0.37
Restricted hobbies^b^	0.57	0.43	0.41	0.41	0.33
(3) Nervous	0.32	0.39	0.65	0.55	0.47
Tension	0.32	0.38	0.69	0.65	0.49
Anxious^a^	0.37	0.39	0.61	0.67	0.45
Depressed	0.41	0.44	0.71	0.62	0.58
(4) Concentration difficulty^a^	0.48	0.47	0.66	0.49	0.48
Remembering difficulty^a^	0.40	0.39	0.70	0.49	0.53
(5) Family life^a^	0.34	0.38	0.55	0.51	0.49
Social life^a^	0.37	0.35	0.50	0.50	0.49
**Symptoms scales**	**Fatigue (6)**	**Nausea and vomitiing (7)**	**Pain (8)**		
(6) Urgency to rest	0.61^d^	0.38	0.44		
Exhausted	0.62	0.41	0.47		
Tired^a^	0.53	0.45	0.59		
(7) Have nausea	0.55	0.56	0.48		
Vomit	0.38	0.56	0.48		
(8) Feel pain^c^	0.49	0.40	0.39		
Miss activites b/c of pain^c^	0.43	0.63	0.39		

a Probable scaling error value of correlation <2SE with another scale, compared with own scale.^7^

b Definite scaling error: value of correlation with another scale >2SE, compared with own scale.

c Did not meet item internal consistency criterion.

d Figures in bold belong to the same subscale.

Of the QLQ-C30 multi-item symptom scales, the two pain items just failed (0.39, each) to meet the IIC criterion, while one item (tiredness) had probable scaling error. The multi-item scales of the BR-23 fared better in IIC and IDV (Table 3). All the items met the IIC criterion of correlation >0.4 with their corresponding scale. There was only one definite scaling error (swelling in arm) and one probable scaling error (flush red face), but these correlated higher only with items that were conceptually logical.

**Table 3:** Item internal consistency and item discriminant validity for QLQ-BR23.

Scale items	Body image (1)	Sexual functioning (2)	Systematic side effects (3)	Breast symptoms (4)	Arm symptoms (5)
(1) Feel unattractive	0.63	0.32	0.54	0.42	0.30
Feel unfeminine	0.71	0.19	0.49	0.37	0.25
Difficulty in looking at self	0.70	0.24	0.41	0.40	0.34
Unsatisfied with body	0.69	0.23	0.47	0.39	0.32
(2) Desire for sex	0.36	0.69	0.39	0.35	0.42
Sexually active	0.24	0.69	0.36	0.35	0.38
(3) Dryness of mouth	0.19	0.20	0.45	0.18	0.26
Taste food differently	0.23	0.23	0.47	0.33	0.27
Eye infection/pain	0.29	0.20	0.49	0.25	0.30
Hair loss	0.36	0.29	0.48	0.27	0.32
Sick/not well	0.47	0.22	0.59	0.37	0.33
Flush/red face^a^	0.52	0.29	0.49	0.42	0.42
Headache	0.55	0.26	0.62	0.39	0.39
(4) Pain affected breast	0.41	0.33	0.37	0.69	0.65
Breast swollen	0.40	0.30	0.44	0.72	0.65
Breast sensitive	0.32	0.32	0.36	0.72	0.56
Skin problems affected breast	0.45	0.33	0.45	0.78	0.59
(5) Pain in arm	0.27	0.35	0.36	0.49	0.55
Swelling in arm^c^	0.30	0.39	0.37	0.67	0.55
Difficulty moving arm	0.39	0.28	0.42	0.73	0.66^c^

a Probabie scaling error: i.e., value of correlation <2SE with another scale, compared with own scale.^7^

b Definite scaling error: value of correlation with another scale >2SE, compared with own scale.

c Figures in bold belong to the same subscale.

### Known-groups validity

For the functional scales of the QLQ-C30, the general population women had significantly higher scores (i.e., better functioning) than the cancer patients, with a difference of at least 10% (effect size >0.5; except emotional scale: ES 0.33, 95%, CI=0.1-0.51). A similar pattern was evident for the multi-item symptom scales of the QLQ-C30, the body image functional scale of the BR-23 (ES 0.80, 95% CI=0.56-1.04), and the multi-item symptom scales of the BR-23 (ES >0.7). Using one-way ANOVA, we found that subjects with advanced disease tended to have worse functioning. This reached significance for the following: role functioning (stage IV <stage II, F=3.8, df=3/335, *P*<.01), diarrhea (stage IV <stages I and II, F=3.5, df=3/338, *P*=.02), and future perspectives (stage III <stages I and II, F=3.5, df=3/329, *P*=.02).

## DISCUSSION

The major limitation of the study is that the cross-sectional design did not allow us to test the sensitivity of the questionnaires to changes in clinical condition. However, our patients had similar demographic characteristics with those of breast cancer clinic populations in Kuwait.^19^,^20^

The data on floor/ceiling effects and missing values indicate that the subjects responded to the full range of options and that the items were broadly acceptable and clear to them.^8^ The problem of reticence in responding to the sexual items was well noted in the original validation study of the BR-23 (consisting of Dutch, Spanish and United States samples), where it was stated that 11% to 14% of patients found one or more of these items to be too personal.^8^ This problem has also been noted in studies using other QOL instruments in the general population^21^ and clinical samples.^22^ Finally, it is to be noted that the item on sexual enjoyment is conditional on having been sexually active, while being upset by hair loss is conditional on having experienced hair loss. Hence, missing values in these items are not good indices of the acceptability of the BR-23 questionnaire.^8^

The Arabic translation of both questionnaires generally met the statistical criteria for the reliability and validity issues investigated. With the exception of the two items of the pain subscale of the QLQ-C30, all the items of both questionnaires met the IIC criterion of >0.4 correlation with the corresponding scale. Coupled with the relatively low number of definite scaling errors, our data support the hypothesized subscales of the QLQ-C30 (except the pain subscale)^13^ and all the subscales of the BR-23.^8^

Our findings indicate that, with the exception of the pain subscale, the hypothesized subscales of the questionnaires are psychometrically sound in the Arab setting.
